# Ameliorating effects of Gö6976, a pharmacological agent that inhibits protein kinase D, on collagen-induced arthritis

**DOI:** 10.1371/journal.pone.0226145

**Published:** 2019-12-06

**Authors:** Tae Won Yoon, Young-In Kim, Hongsik Cho, David D. Brand, Edward F. Rosloniec, Linda K. Myers, Arnold E. Postlethwaite, Karen A. Hasty, John M. Stuart, Ae-Kyung Yi

**Affiliations:** 1 Department of Microbiology, Immunology and Biochemistry, University of Tennessee Health Science Center, Memphis, Tennessee, United States of America; 2 Department of Pediatrics, University of Tennessee Health Science Center, Memphis, Tennessee, United States of America; 3 Department of Orthopedic Surgery and Biomedical Engineering, University of Tennessee Health Science Center, Memphis, Tennessee, United States of America; 4 Department of Medicine, University of Tennessee Health Science Center, Memphis, Tennessee, United States of America; 5 Veterans Affairs Medical Center-Memphis, Memphis, Tennessee, United States of America; Macau University of Science and Technology, MACAO

## Abstract

Toll-like receptor (TLR) signaling can contribute to the pathogenesis of arthritis. Disruption of TLR signaling at early stages of arthritis might thereby provide an opportunity to halt the disease progression and ameliorate outcomes. We previously found that Gö6976 inhibits TLR-mediated cytokine production in human and mouse macrophages by inhibiting TLR-dependent activation of protein kinase D1 (PKD1), and that PKD1 is essential for proinflammatory responses mediated by MyD88-dependent TLRs. In this study, we investigated whether PKD1 contributes to TLR-mediated proinflammatory responses in human synovial cells, and whether Gö6976 treatment can suppress the development and progression of type II collagen (CII)-induced arthritis (CIA) in mouse. We found that TLR/IL-1R ligands induced activation of PKD1 in human fibroblast-like synoviocytes (HFLS). TLR/IL-1R-induced expression of cytokines/chemokines was substantially inhibited in Gö6976-treated HFLS and PKD1-knockdown HFLS. In addition, serum levels of anti-CII IgG antibodies, and the incidence and severity of arthritis after CII immunization were significantly reduced in mice treated daily with Gö6976. Synergistic effects of T-cell receptor and TLR, as well as TLR alone, on spleen cell proliferation and cytokine production were significantly inhibited in the presence of Gö6976. Our results suggest a possibility that ameliorating effects of Gö6976 on CIA may be due to its ability to inhibit TLR/IL-1R-activated PKD1, which might play an important role in proinflammatory responses in arthritis, and that PKD1 could be a therapeutic target for inflammatory arthritis.

## Introduction

Rheumatoid arthritis (RA) is a chronic and crippling autoimmune disease with destructive inflammation in joints that affects 1 to 2% of the population. Although the factors that initiate and sustain RA are unknown, an exaggerated innate immune response involving the joint develops early in RA and may serve as a key pathogenic mechanism that initiates synovial inflammation and subsequently leads to development of an autoimmune reaction to joint-specific antigens in genetically susceptible individuals [1]. We recognize that joint inflammation may be related to multiple autoantigens and that control of the autoimmune reaction to a single autoantigen may be inadequate to completely control the disease. We also recognize that autoimmune reactions may be mediated through nontraditional pathways, such as a self-augmenting reaction that involves signaling through receptors in the innate immune system, especially Toll-like receptors (TLRs). In support of this concept is the finding that TLR agonists, including proteoglycans (PGN), bacterial DNA, and autologous ligands, have been detected in rheumatoid synovium [2]. TLRs link innate and adaptive immunity by promoting the expression of cytokines, chemokines, and co-stimulatory molecules in antigen-presenting cells (APCs) that contribute to the accumulation of various immune cells to the site of inflammation, and by driving lymphocyte activation and differentiation. Although TLRs are primarily involved in the innate immune response to microbial pathogens [3], they also contribute to sterile inflammation by sensing the endogenous molecules [called “danger signals”] that are generated during tissue damage or inflammation [4–6]. Indeed, both microbial and endogenous TLR ligands have repeatedly been used to induce arthritis in susceptible animals [7–9], and blocking of TLRs or TLR-signaling modulators ameliorates progression of the disease in experimental models of arthritis [10, 11], and inhibits spontaneous production of proinflammatory cytokines and matrix metalloproteinases (MMPs) by RA synovial cells [12–14]. In addition, TLRs share part of their signal transduction pathways with the receptors of IL-1 and IL-18 (which are known as critical proinflammatory cytokines that are involved in the pathogenesis of RA). In theory, interruption of the common TLR/IL-1R signaling sequence in inflamed joints may provide an effective therapy. Several investigators have recently identified TLR/IL-1R signaling as an important therapeutic target in treating autoimmune diseases [11, 15, 16].

Previously, we found that protein kinase D1 (PKD1), a serine/threonine protein kinase, is activated by all TLRs and IL-1R that transduce their signal through myeloid differentiation factor 88 (MyD88) in both humans and mice [17, 18]. Upon cognate ligand binding, TLR/IL-1R recruits PKD1 to the receptor complex [18]. Activation of PKD1 in the receptor complex of TLR/IL-1R depends on IRAK4, IRAK4 kinase activity and IRAK1 but is independent of TRAF6 [17, 19]. IRAK4 kinase activity is also important PKD1 is required for ubiquitination of TRAF6 and all subsequent downstream events, including activation of TAK1, MAPKs, and NF-κB, and expression of proinflammatory mediators, except for type I IFNs, in humans and mice [17, 18]. In addition, we found that TLR/IL-1R ligand-induced activation of PKD1 and expression of proinflammatory mediators are inhibited by a pharmacological agent that inhibits PKD, such as Gö6976 and CRT0066101, but not by an agent that inhibits PKC, such as Gö6983 [17–21]. These findings imply the possibility that PKD1 could be one of the regulatory factors that contributes to the development of proinflammatory reactions in the joints in susceptible individuals and that PKD inhibitors could be valuable therapeutic options for controlling arthritis. However, it is completely unknown at this point whether PKD1 plays a protective or a detrimental role during the course of any form of arthritis. In the present study we investigated whether treatments with the pharmacological agent Gö6976, which can suppress TLR/IL-1R-induced PKD1 activity, alter the pathogenic course of an experimental arthritis.

## Materials and methods

### Mice

Type II collagen (CII)-specific T cell receptor (TCR) and human leukocyte antigen (HLA) alleles DRB1*0101 (DR1; a haplotype susceptible to RA) expressing double transgenic mice (TCR/DR1 dtg) [22] and HLA-DR1 expressing transgenic mice (DR1 tg) [23] were bred and maintained in a pathogen free facility. All animal care and housing requirements set forth by the National Institutes of Health Committee on the Care and Use of Laboratory Animals of the Institute of Laboratory Animal Resources were followed, and animal protocols were reviewed and approved by the University of Tennessee Institutional Animal Care and Use Committee and Veterans Affairs Medical Center-Memphis Institutional Animal Care and Use Committee.

### Cells and culture conditions

TCR/DR1 dtg at 12 to 16 wk of age were used as a source of splenic leukocytes. Briefly, mice were killed by cervical dislocation. Single cell suspensions were aseptically prepared from the spleens of the mice. Red blood cells were lysed and splenic leukocytes were isolated after centrifugation over Lympholyte M (Cedarlane Laboratories, Hornby, Canada). Isolated splenic cells were cultured in RPMI 1640 supplemented with 10% (v/v) heat-inactivated fetal bovine serum (FBS), 1.5 mM L-glutamine, 50 mM 2-ME, 100 units/ml penicillin, and 100 μg/ml streptomycin at 37°C in a humidified incubator with 5% CO_2_. Human fibroblast-like synoviocytes (HFLS) isolated from synovial tissues obtained from donors without rheumatoid arthritis (HFLS-N; HFLS-Normal) or with rheumatoid arthritis (HFLS-RA) were purchased from Cell Applications, Inc., (San Diego, CA). HFLS were cultured in human synoviocytes growth medium (Cell Applications, Inc.) at 37°C in a humidified incubator with 5% CO_2_ and used between passages 5 to 6.

### Synthetic CII peptide, Toll-like receptor (TLR) ligands, and reagents

Synthetic CII peptide sequence TGE[hyp]GIAGFKGEQGPKGE[hyp]G that containing the immunodominant determinant sequence of both bovine and human CII (GIAGFKGEQGPKGEB) [22] was purchased from ABI Scientific Inc. (Sterling, VA). Peptidoglycan (PGN), Pam_3_CSK_4_ (Pam3), and poly(I:C) (PIC) were purchased from InvivoGen (San Diego, CA). Ultra-pure lipopolysaccharide (LPS; from *Escherichia coli* 0111:B4) was purchased from List biological laboratories, Inc. (Campbell, CA). Human recombinant IL-1β was purchased from Life Technologies (Carlsbad, CA). PMA was purchased from Sigma-Aldrich Co. (St. Louis, MO). Gö6976 and Gö6983 were purchased from Calbiochem (La Jolla, CA).

### Small interfering RNA (siRNA) transfection

HFLS were plated at 2 x 10^6^ cells/10 ml in a 100-mm Petri dish and incubated overnight and then transfected with 100 nM of non-target siRNA or human PKD1-specific SMARTpool siRNAs (Dharmacon, Lafayette, CO) using Lipofectamine (Invitrogen, Carlsbad, CA) according to the manufacturer’s protocol.

### RT-PCR, enzyme-linked immunosorbent assay (ELISA), multiplex sandwich immunoassay and Western blot assay

Levels of the selected cytokine and chemokine genes in cells, concentrations of the selected cytokines and anti-CII antibodies, and levels or phosphorylation status of specific proteins in whole cell extracts were analyzed by RT-PCR, ELISA, multiplex sandwich immunoassay and Western blot assay, respectively, as described previously [18, 20, 24]. Actin was used as a loading control for all RT-PCR and Western blot assays. All primers for RT-PCR were purchased from Integrated DNA Technologies, Inc. (Coralville, IA). Primers used for human genes are: PKD1 (F:5’GACCAGAAGTTCCCTGAATG3’, R: 5’ATTCAGACCACACCCTTCAC3’), IL-6 (F:5’ACTCACCTCTTCAGAACGAA3’, R:5’CTCAAACTCCAAAAGACCAG3’), IL-8 (F:5’CACCCCAAATTTATCAAAGA3’, R:5’TCAAAAACTTCTCCACAACC3’), MIP2 (F:5’CGAGGAGAAGATGGTTATCA3’, R:5’TATTCTTCGTAGAACCTGCG3’), and β-actin (F:5’GTGGGCGCCCCAGGCACCA3’, R:5’CTCCTTAATGTCACGCACGATTTC3’). All recombinant murine cytokines and antibodies (Abs) specific for murine cytokines were purchased from eBioscience (San Diego, CA). Murine cytokine multiplex kits were purchased from EMD Millipore (Billerica, MA). Isotype-specific anti-collagen IgG ELISA kits were purchased from Chondrex, Inc. (Redmond, WA). Abs specific for PKD, IRAK1, TRAF6 or actin were purchased from Santa Cruz Biotechnology, Inc. (Santa Cruz, CA) or (Millipore, Billerica, MA). Phospho-specific anti-PKD Abs (pPKDs916) were purchased from Cell Signaling (Beverly, MA).

### Cell proliferation assay

Spleen cells (1 x 10^5^ cells/300 μl/well in 96-well plate) were pretreated with DMSO (1% v/v; vehicle), Gö6976 (250 or 500 ng/ml), or Gö6983 (250 or 500 ng/ml) for 30 min and then stimulated with media, synthetic CII peptide (10 μg/ml), and/or Pam_3_CSK_4_ (500 ng/ml) for designated time periods at 37°C / 5% CO_2_. One μCi of [^3^H]thymidine (New England Nuclear, Boston, MA, USA) was added to each well and then incubated for the designated time period as indicated in the Fig legend. Cells were harvested onto glass fiber filters and were counted on a Matrix 96 direct ionization B-counter (Packard Instrument Company, Meriden, CT, USA).

### Induction of arthritis by immunization with CII

Native CII was solubilized from bovine articular cartilage by limited pepsin digestion and purified as described previously [25–27]. CII was dissolved in 0.01M acetic acid and emulsified with an equal volume of complete Freund’s adjuvant (CFA; Sigma-Aldrich Co.) as described previously [26]. The resulting emulsion was injected into the base of the tail subcutaneously. Each mouse received a total volume of 0.05 ml containing 100 μg mycobacterium tuberculosis and 100 μg of CII antigen. In some experiments, mice were administered with vehicle (7.6% v/v DMSO in saline), Gö6976 (inhibits PKD: 2.3 mg/kg body weight), or Gö6983 (inhibits PKC: 2.3 mg/kg body weight) by intraperitoneal injection once a day started at the day of CII immunization. The *in vivo* dose for Gö6976 and Gö6983 was selected based on the previously optimized effective dose of the inhibitor (the dose at which Gö6976 inhibits proinflammatory responses to a TLR ligand, *Saccharopolyspora rectivirgula*, or Group B streptococci *in vivo*, while Gö6983 does not) [17–20].

### Clinical evaluation of arthritis

The presence of joint inflammation in mice was evaluated daily for the duration of the experiment by two blinded independent investigators. Arthritis was defined as visible erythema, swelling, or joint rigidity of at least one joint. To evaluate the intensity (severity) of arthritis, the following clinical scoring system [26, 28] was used for each limb: 0 point, normal paw; 1 point, one toe inflamed or swollen; 2 points, more than one toe but not the entire paw inflamed and swollen or mild swelling of entire paw; 3 points, entire paw inflamed and swollen; 4 points, very inflamed and swollen or ankylosed paw. Paws were individually scored and total maximum score for each mouse is 16. The mean arthritis severity for each experimental group was constructed by dividing the total severity score by the number of animals used in each experimental group. The percentage of arthritic paws was calculated by dividing the total number of arthritic paws by the total number of paws in each experimental group times 100. The mean number of arthritic paws was calculated by dividing the total number of arthritic paws by the number of animals used in each experimental group. The arthritis severity per arthritic mouse was constructed by dividing the total severity score by the number of arthritic animals in the each experimental group. The number of arthritic paws per arthritic mouse was calculated by dividing the total number of arthritic paws by the number of arthritic animals in the treatment group.

### Histological studies

Joints were removed, fixed in 10% formalin for 24 hr, decalcified in 5% trichloroacetic acid for 7 days, dehydrated, embedded in paraffin, sectioned at 5 μm, and stained with hematoxylin and eosin (H&E; to determine inflammatory cell influx and chondrocyte death) or safranin O and Fast Green (S&F; to determine proteoglycan depletion and cartilage and bone destruction). Digital images of whole H&E- or S&F-stained slides were captured using the Aperio ScanScope® XT Slide Scanner (Aperio Technologies, Inc. Vista CA) system. Histopathological features of arthritis were examined by two blinded independent investigators. A histopathologic score assigned to paws was based upon the extent of inflammation, pannus formation, cartilage damage, and bone erosion, each using a scale of 0 (none to minimal) to 3 (the most severe) [29, 30]. At least 4 fields/paw at 10x of the original scanning were scored and averaged. Paws were individually scored and total maximum score for each paw is 12. Two hind paws/mouse were analyzed. The mean arthritic histopathologic score for each experimental group was constructed by dividing the sum of the total score of each paw in the group by the number of paws in each experimental group.

### Detection of matrix metalloproteinase (MMP) activity in joints

MMP activities in joints of mice were detected and analyzed as previously described [31, 32] with a minor modification. Briefly, mice were injected retro-orbitally with 50 μl of a solution containing MMPSense® 750 FAST Fluorescent Imaging Agent (750F) (Perkin Elmer, Hopkinton, MA). MMPSense® 750 is an optically silent MMP substrate that produces fluorescent signal after cleavage by a broad range of MMPs including MMP 2, 3, 7, 9, 12, and 13 [33, 34]. Twenty four hours later, mice were anesthetized and then scanned using IVIS® Lumina XR System (Perkin Elmer, Hopkinton, MA) with a high range filter set. The excitation/emission wavelengths used for 750F were 745 nm/800 nm. Living Image 4.0 software was used to calculate the flux radiating omni-directionally from the region of interest (ROI) in each joint. ROI was defined as the area where the foot and leg meet, and knee area where femur, patella and tibia meet. This area consists of the following four joints: knee joint, true ankle joint, subtalar joint, and inferior tibiofibular joint. Fluorescence intensities were quantified within the ROI by using Living Image 4.0 software. Each sample was quantified thrice and the average was taken. The average of each sample per group represented the mean value of each group. Background fluorescence was removed by subtracting the fluorescence of the null or background capture area (consisting of muscle and skin tissue) from each articular reading. Calculations are represented graphically as radiant efficiency (photons/s/cm^2^/str)/(μW/cm^2^).

### Statistical analysis

Data were expressed as mean ± SD. The differences between the control and experimental groups were evaluated using two-tailed Student’s *t*-test. Statistical differences with *p* < 0.05, *p* < 0.005, *p* < 0.0005, and *p* < 0.00005 are indicated as *, **, #, and ##, respectively, and considered significant.

## Results

### PKD1 is essential for TLR/IL-1R-induced expression of proinflammatory mediators in human primary fibroblast-like synoviocytes

**Fig 1:**
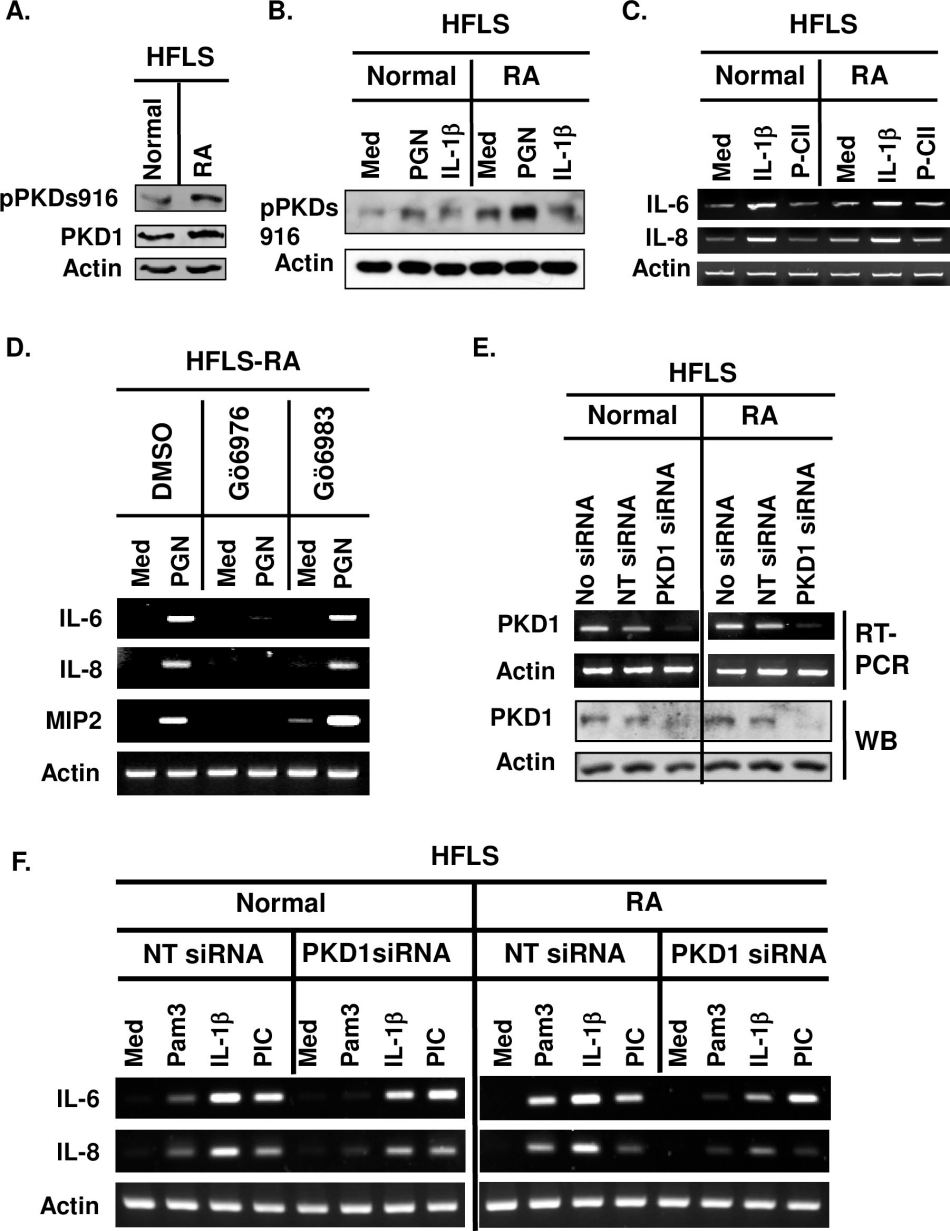
PKD1 can be activated and plays an essential role in both spontaneous and TLR/IL-1R-induced expression of proinflammatory mediators in human synoviocytes. **(A)** Protein levels and phosphorylation status of PKD in human primary fibroblast-like synoviocytes (HFLS) from a normal donor (HFLS-N) and from a patient with RA (HFLS-RA) were detected by Western blot. **(B)** HFLS-N and HFLS-RA were stimulated with media (med), TLR2 ligand peptidoglycan (PGN; 50 μg/ml), or human recombinant IL-1β (10 ng/ml) for 45 min. Protein levels and phosphorylation status of PKD were detected by Western blot. **(C)** HFLS-N and HFLS-RA were stimulated with media (med), IL-1β (10 ng/ml), or synthetic CII peptide (P-CII; 50 μg/ml) for 4 hr. Levels of IL-6 and IL-8 mRNA were analyzed by RT-PCR. **(D)** HFLS-RA were pretreated with vehicle (1% v/v DMSO), Gö6976 (500 ng/ml) or Gö6983 (500 ng/ml) for 1hr and then stimulated with PGN (10 μg/ml) for 4 hr. Levels of the indicated cytokine and chemokine mRNA were analyzed by RT-PCR. **(E, F)** HFLS-N and HFLS-RA were transiently transfected with non-target siRNA (NT siRNA; control) or PKD1-specific siRNA (PKD1 siRNA; PKD1-knockdown). mRNA levels (E-top panel) and protein levels (E-bottom panel) of PKD1 were detected by RT-PCR and Western blot, respectively. Control and PKD1-knockdown HFLS were stimulated with Pam_3_CSK_4_ (Pam3; 500 ng/ml), IL-1β (10 ng/ml), or TLR3 ligand PIC (50 μg/ml) for 4 hr. Messenger RNA levels of the indicated gene were analyzed by RT-PCR. All experiments were repeated two to three times with similar results. S1 Fig shows uncropped blot and gel scans.

We previously found that PKD1 is required for MyD88-dependent expression of proinflammatory mediators in humans and mice [17–20]. However, the role of PKD1 in TLR/IL-1R-mediated proinflammatory responses in human synoviocytes is currently unknown. We investigated whether TLR/IL-1R ligands can activate PKD1 and whether PKD1 plays a role in TLR/IL-1R-mediated proinflammatory gene expression in human fibroblast-like synoviocytes isolated from donor without rheumatoid arthritis (HFLS-N) or with rheumatoid arthritis (HFLS-RA). As shown in Fig 1A, both HFLS-N and HFLS-RA expressed PKD1. Increased phosphorylation of PKD and expression of IL-6 and IL-8 in response to TLR/IL-1R ligands were detected in both HFLS-N and HFLS-RA (Fig 1B and 1C). Synthetic CII peptide containing the immunodominant determinant sequence of human CII (P-CII) did not induce expression of IL-6 and IL-8 above the basal levels in both FHLS-N and HFLS-RA (Fig 1C). These results indicate that PKD1 can be activated by signals mediated through TLR/IL-1R in synovial cells in human. These results also implicate that PKD1 could contribute to the exaggerated proinflammatory responses seen in arthritic joints.

Because we found in our previous studies [17–21] that inhibition of PKD by Gö6976, but not inhibition of PKC by Gö6983, resulted in inhibition of TLR/IL-1R ligand-induced expression of proinflammatory mediators, we further investigated whether Gö6976 can suppress proinflammatory reactions in response to TLR/IL-1R ligands in HFLS-RA. As demonstrated in Fig 1D, PGN failed to induce expression of the selected cytokines and chemokines (IL-6, IL-8, and MIP2) in HFLS-RA pretreated with Gö6976. In contrast, PGN-induced expression of those cytokines and chemokines in HFLS-RA was not affected by the presence of Gö6983. Taken together, these results suggest that PKD family members (presumably PKD1) play a critical role in the TLR/IL-1R-induced expression of cytokines and chemokines in HFLS-RA.

Next, to further confirm the findings with Gö6976 that PKD1 is required for the TLR/IL-1R-mediated expression of cytokines and chemokines in HFLS-RA, we silenced PKD1 expression by transiently transfecting HFLS-N and HFLS-RA with non-target siRNA (NT siRNA; control cells) or PKD1-specific siRNA (PKD1-siRNA; PKD1-knockdown cells). Expression of PKD1 mRNA and protein was substantially inhibited in PKD1-knockdown cells compared to the control NT-siRNA cells (Fig 1E). These results demonstrate that PKD1-siRNA specifically and effectively knockdown PKD1 expression in these cells. Using these PKD1-knockdown cells, we further investigated whether PKD1 plays a role in TLR/IL-1R-induced expression of cytokines in HFLS-N and HFLS-RA. We found that TLR2 ligand Pam_3_CSK_4_ (Pam3)- or IL-1β-induced expression of cytokines (IL-6 and IL-8) was substantially inhibited in PKD1-knockdown HFLS-N and PKD1-knockdown HFLS-RA (Fig 1F). In contrast, TLR3 ligand PIC-induced expression of cytokines was not inhibited in PKD1-knockdown HFLS-N and PKD1-knockdown HFLS-RA. These results indicate a critical regulatory role of PKD1 in MyD88-dependent TLR/IL-1R-mediated expression of proinflammatory mediators in HFLS-N and HFLS-RA. Collectively, these results imply that PKD1 might be one of the key factors that modulate pathogenesis of certain types of human arthritis, including RA and that activity of TLR/IL-1R-induced PKD1 can be suppressed by Gö6976.

### Gö6976 treatments significantly reduce development and progression of collagen-induced arthritis

**Fig 2:**
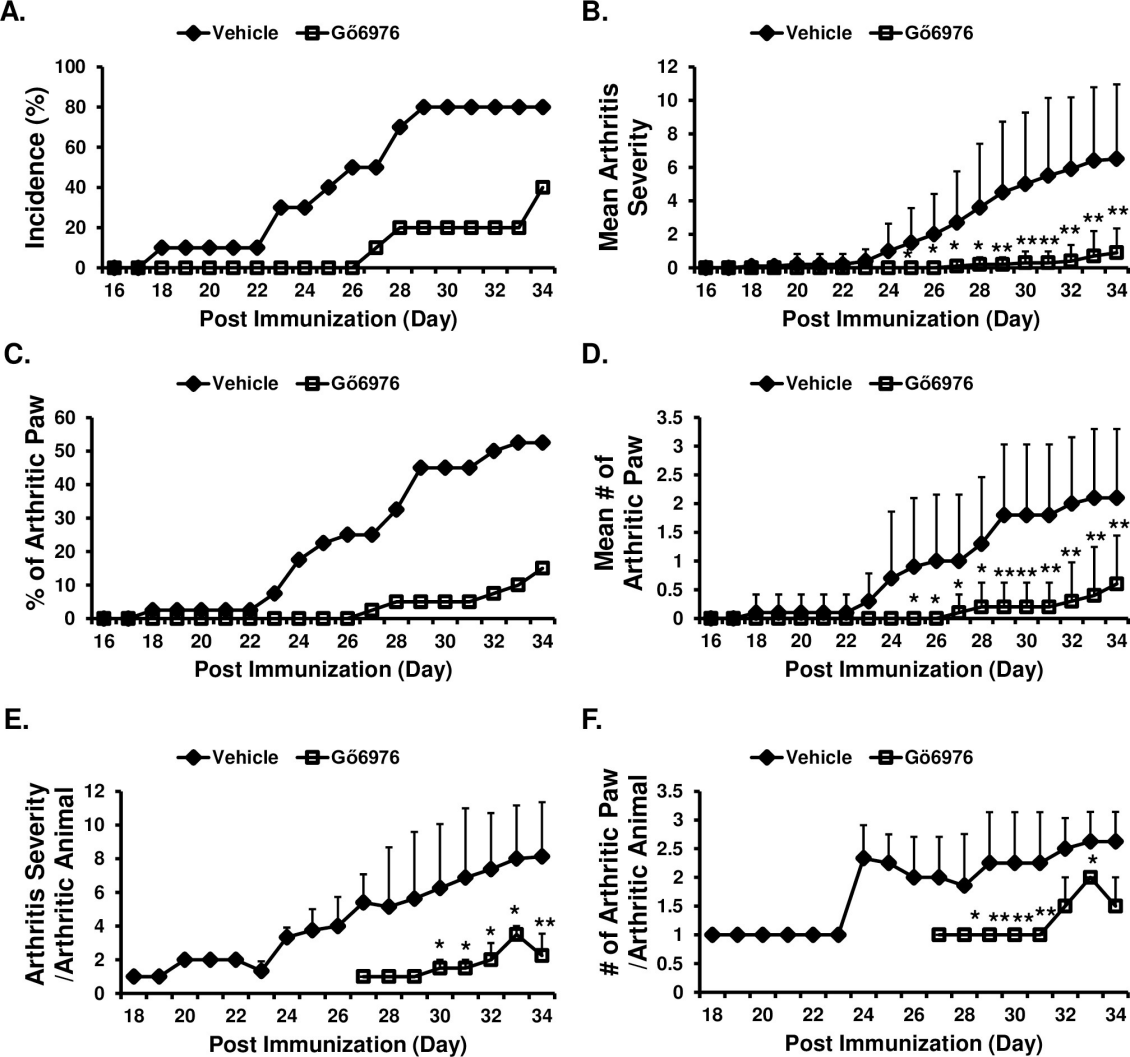
Effects of Gö6976 on the development and progression of CIA. HLA-DR1 mice were immunized with bovine CII and treated daily with vehicle (n = 10) or Gö6976 (n = 10) for 34 days starting at the day of CII immunization. Mice were observed daily and development of arthritis was assessed. **(A)** Incidence of arthritis. **(B)** Mean arthritis severity. Data represent the mean ± SD. *, *p* < 0.05; **, *p* < 0.005. **(C)** The % of arthritic paws. **(D)** Mean number of arthritic paws in a mouse. Data represent the mean ± SD. *, *p* < 0.05; **, *p* < 0.005. **(E)** Arthritis severity score in an arthritic mouse. Data represent the mean ± SD. *, *p* < 0.05; **, *p* < 0.005. **(F)** Number of arthritic paws in an arthritic mouse. Data represent the mean ± SD. *, *p* < 0.05; **, *p* < 0.005. Experiments were repeated four times with similar results.

Because our results showed that PKD1 is activated and essential for the TLR/IL-1R-induced cytokine and chemokine expression in HFLS isolated from patients with RA, we thought that a pharmacological agent that can inhibit TLR/IL-1R-mediated PKD1 activation may have ameliorating effects on arthritis. Therefore, we investigated whether treatments with Gö6976 can affect the development and progress of collagen-induced arthritis (CIA; an experimental animal model for human RA). CII-immunized HLA-DR1 mice were treated daily with vehicle or Gö6976 and the presence of joint inflammation and the severity of arthritis were examined. As shown in Fig 2A, compared to the vehicle-treated control mice, the incidence of arthritis was substantially reduced and the initial development of arthritis was greatly delayed in mice treated with Gö6976. Severity of arthritis (Fig 2B), percentage of total arthritic paws in the treatment group (Fig 2C), and average number of arthritis paws in a mouse (Fig 2D) were also significantly reduced in mice treated with Gö6976. We further analyzed whether there is any difference in the vehicle-treated group versus the Gö6976 group for arthritis severity and/or number of arthritis paws in mouse that developed arthritis. As demonstrated in Fig 2E and 2F, even if a mouse developed arthritis, Gö6976-treated mouse developed significantly less severe arthritis and significantly fewer number of arthritic paws compared to the vehicle-treated control mouse that had developed arthritis. These results demonstrate that treatments with a pharmacological agent that inhibits PKD1 can significantly delay onset of the disease and ameliorate the disease outcomes of CIA.

### Gö6976 treatments significantly inhibit anti-CII antibody production and subsequent joint destruction

**Fig 3:**
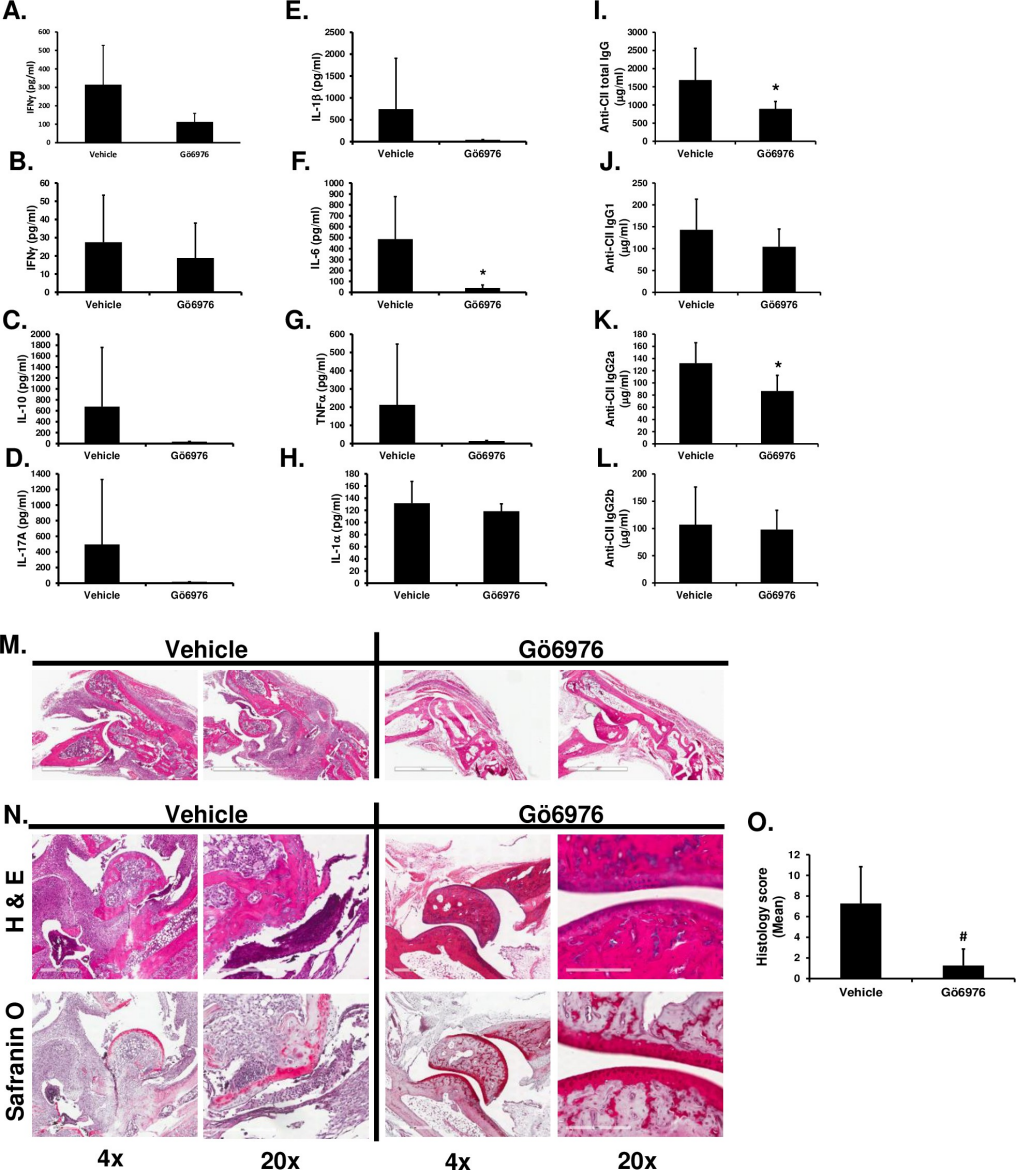
Effects of Gö6976 treatments on the anti-CII IgG antibody production, joint inflammation and bone destruction in HLA-DR1 mice immunized with CII. HLA-DR1 mice were immunized with bovine CII and treated daily with vehicle (n = 4) or Gö6976 (n = 5) for 34 days starting at the day of CII immunization. Blood (at day 11 and day 35) and joints (at day 35) were harvested. **(A—H)** Levels of the selected cytokines in serum isolated at day 11 (A) and at day 35 (B—H) were analyzed by multiplex sandwich immunoassay. Data represent the mean concentration (pg/ml) ± SD. *, *p* < 0.05. **(I–L)** Levels of total anti-CII IgG antibodies (I), anti-CII IgG1 antibodies (J), anti-CII IgG2a antibodies (K) and anti-CII IgG2b antibodies (L) in serum isolated at day 35 were analyzed by isotype-specific anti-CII IgG ELISA. Data represent the mean concentration (μg/ml) ± SD. *, *p* < 0.05. **(M)** Representative H&E stained joint tissue section (1.5 x of the original objective; white scale bar = 2 mm). **(N)** Representative H&E (top) or safranin O (bottom) stained joint tissue sections. White scale bar = 600 μm at 4 x of the original objective. White scale bar = 200 μm at 20 x of the original objective. **(O)** Histology score (vehicle: n = 8 paws, Gö6976: n = 10 paws). Data represent the mean ± SD. #, *p* < 0.0005. Experiments were repeated two to four times with similar results.

To further investigate whether Gö6976 treatments alter systemic cytokine production and the pathogenic anti-CII antibody production that might contribute to the joint destruction in CIA, HLA-DR1 mice were immunized with CII and treated daily with Gö6976. As shown in Fig 3A–3G, the serum levels of IFNγ, IL-10, IL-17A, IL-1β, IL-6 and TNFα were lower in the Gö6976-treated group compared to the vehicle-treated group. However, differences were not statistically significant. The serum levels of IL-1α were not different in CIA mice treated with vehicle versus Gö6976 (Fig 3H). As demonstrated in Fig 3I, serum levels of anti-CII total IgG antibody were significantly lower in CII-immunized HLA-DR1 mice treated with Gö6976 compared to those in mice treated with vehicle. Isotype-specific IgG analysis of anti-CII antibodies showed that serum levels of IgG2a isotype anti-CII antibody were significantly reduced in mice treated with Gö6976, while serum levels of IgG1 isotype and IgG2b isotype anti-CII antibodies in mice treated with Gö6976 were comparable to those in mice treated with vehicle (Fig 3J–3L).

To further investigate the effects of Gö6976 on joint inflammation and destruction, histopathological features of arthritis in joints of CII-immunized HLA-DR1 mice treated with or without Gö6976 were analyzed. As demonstrated in Fig 3M–3O, CII-immunized HLA-DR1 mice treated with vehicle showed severe deformation of foot ankle and joints. These mice showed substantial leukocyte infiltration in joint spaces and severe pannus formation, cartilage damage, and bone erosion. Compared to these control mice, CII-immunized HLA-DR1 mice treated with Gö6976 showed significantly reduced joint inflammation (assessed by leukocyte infiltration in joint spaces), pannus formation, cartilage damage, and bone erosion (Fig 3M–3O). Taken together, these results demonstrated that Gö6976 treatments have ameliorating effects on the development and progress of CIA.

### Removal of Gö6976 from treatment protocol after successful inhibition of arthritis development does not further aggravate progression of CIA

**Fig 4:**
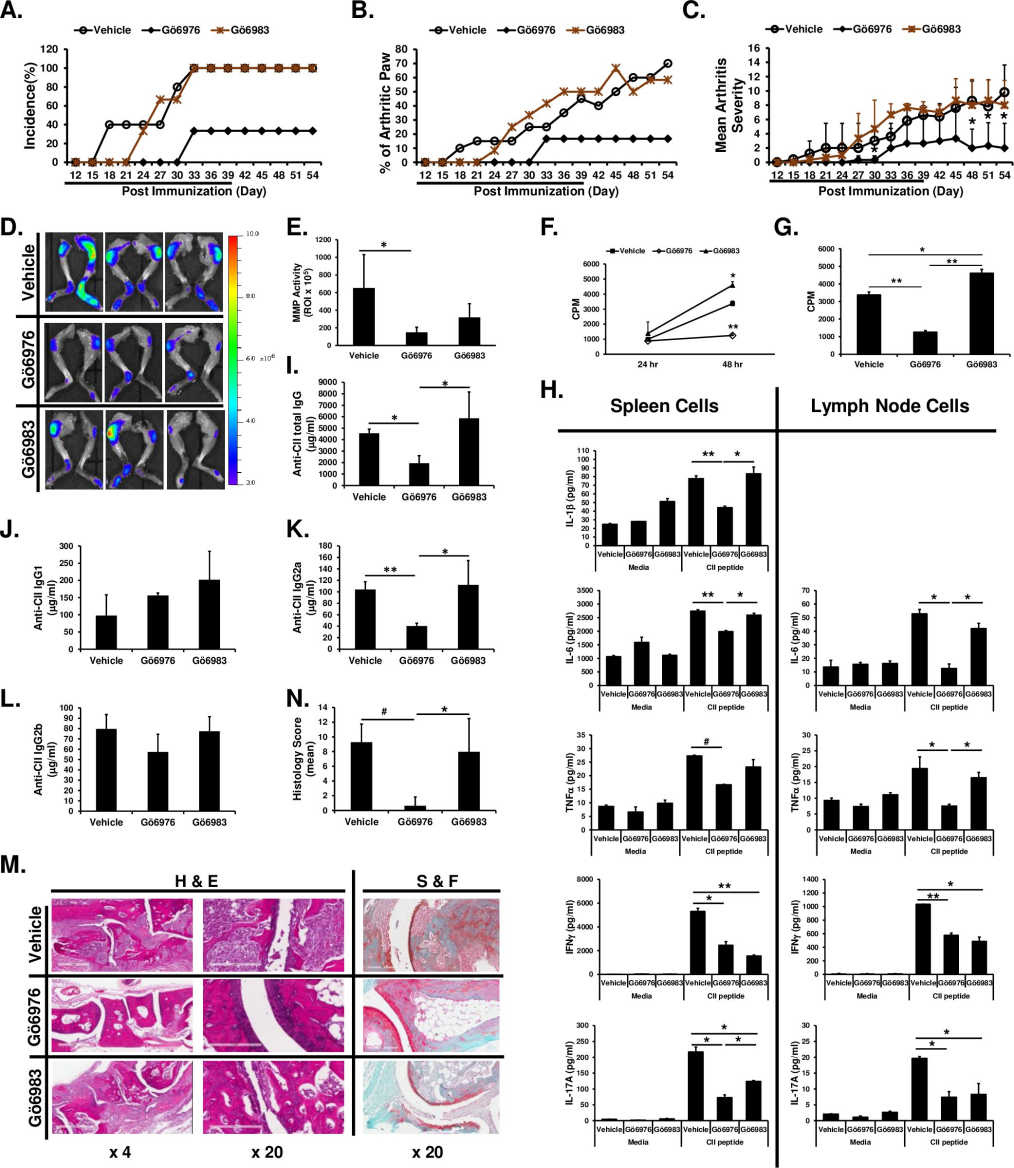
Differential effects of Gö6976 and Gö6983 on CIA. HLA-DR1 mice were immunized with bovine CII and treated daily with vehicle (n = 5), Gö6976 (n = 3), Gö6983 (n = 3) for 39 days (indicated as underline) starting at the day of CII immunization. Mice were observed daily and development of arthritis was assessed up to 54 days post immunization. **(A)** Incidence of arthritis. **(B)** The % of arthritic paws. **(C)** Mean arthritis severity score. Data represent the mean ± SD. *, *p* < 0.05. **(D—E)** At day 54, mice were injected i.v. with MMPSense® 750 FAST Fluorescent Imaging Agent (750F). Twenty four hours later, mice were anesthetized and then scanned using IVIS® Lumina XR System (D). Fluorescence intensities were quantified within ROI by using Living Image 4.0 software (E). Calculations are represented graphically as radiant efficiency (photons/s/cm^2^/str)/(μW/cm^2^). Data represent the mean of the group ± SD. *, *p* < 0.05. **(F-N)** At day 55, inguinal lymph nodes, spleens, blood, and hind paws were harvested. **(F, G)** Lymph node cells were incubated in 96-well plate (8 x 10^5^ cells/200 μl/well) for 43 hrs. One μCi of [^3^H]thymidine was added to each well, and the cells were harvested after an additional 5 hr of culture. Data present the mean (cpm) ± SD. *, *p* < 0.05; **, *p* < 0.005. **(H)** Spleen cells and lymph node cells were stimulated with media or synthetic CII peptide (10 μg/ml) for 24 hr (for IL-6 in spleen cell culture), 48 hr (for IFNγ and IL-17A in both spleen cell and lymph node cell culture), or 72 hr (for IL-1β and TNFα in both spleen cell and lymph node cell culture, and IL-6 on lymph node cell culture). The levels of cytokines in the culture supernatants were analyzed by multiplex sandwich immunoassay. Data represent the mean concentration (pg/ml) ± SD. *, *p* < 0.05; **. **(I–L)** Levels of total serum anti-CII IgG antibodies (I), anti-CII IgG1 antibodies (J), anti-CII IgG2a antibodies (K) and anti-CII IgG2b antibodies (L) were analyzed by isotype-specific anti-CII IgG ELISA. Data represent the mean concentration (μg/ml) ± SD. *, *p* < 0.05; **, *p* < 0.005. **(M)** Representative H&E or S&F stained joint tissue sections at day 55 post immunization. White scale bar = 600 μm at 4 x of the original objective. White scale bar = 200 μm at 20 x of the original objective. **(N)** Histology score (vehicle: n = 10 paws, Gö6976: n = 6 paws, Gö6983: n = 6 paws). Data represent the mean ± SD. *, *p* < 0.05; #, *p* < 0.0005.

We further investigated whether Gö6983, which inhibits conventional PKC but does not inhibit PKD, has similar effects with Gö6976, which inhibits conventional PKC and PKD, on development of CIA and whether HLA-DR1 mice will develop arthritis later if Gö6976 treatments cease after the successful inhibition of arthritis development. HLA-DR1 mice were immunized with CII and treated daily with vehicle, Gö6976, or Gö6983 for 39 days and then mice were maintained without drug treatment up to 54 days post CII immunization. As shown in Fig 4A–4C, initial development of arthritis was slightly delayed in mice treated with Gö6983, but overall visible clinical symptoms of arthritis (based on arthritis incidence, number of arthritic limbs, and arthritis severity score) as the disease progressed were similar between mice in the vehicle-treated group and in the Gö6983-treated group. In contrast, the initial development of arthritis was greatly delayed and the incidence of arthritis was substantially reduced in CII-immunized HLA-DR1 mice treated with Gö6976 (Fig 4A and 4B). In addition, severity of arthritis was significantly reduced in mice treated with Gö6976 (Fig 4C). Moreover, ceasing Gö6976 treatment after 39 days (indicated with an underline in the Fig 4A–4C) did not further increase incidence and severity of arthritis (Fig 4A–4C).

Enzyme activity of matrix metalloproteinases (MMPs) in joint cavities positively correlate with the disease severity [32]. Thus, upon termination of the experiment, we evaluated MMP activity in the joints of CII-immunized HLA-DR1 mice treated with vehicle, Gö6976, or Gö6983. As shown in Fig 4D and 4E, fluorescent signals (indicating MMP enzyme activities) were drastically increased in joints of the CII-immunized HLA-DR1 mice treated with vehicle. MMP activities in the joints were significantly reduced in the mice treated with Gö6976 compared to those in the mice treated with vehicle. Although it did not reach statistical significance, MMP activities in the joints of the CII-immunized HLA-DR1 mice treated with Gö6983 were greatly reduced, indicating a possibility that some inflammatory processes in the course of CIA are also mediated through activation of PKC family members, such as PKCα and β, in addition to PKD. However, MMP activities in the joints of the mice treated with Gö6976 were substantially lower than those in the mice treated with Gö6983, indicating a critical role of PKD in proinflammatory reactions in the joints during the pathogenic course of CIA.

The inguinal lymph nodes were also removed from the experimental mice at day 55 after the CII-immunization and cells were cultured to analyze spontaneous cell proliferation *ex vivo*. As shown in Fig 4F and 4G, spontaneous cell proliferation *ex vivo* was found in the lymph node cells isolated from the CII-immunized HLA-DR1 mice treated with vehicle or Gö6983. In contrast, significantly decreased cell proliferation was found in the lymph node cells isolated from the CII-immunized HLA-DR1 mice treated with Gö6976. To analyze cytokine response to the immunized antigen (CII), spleen cells and inguinal lymph node cells isolated from these CII-immunized HLA-DR1 mice treated with vehicle, Gö6976, or Gö6983 were stimulated with antigenic synthetic CII peptide *ex vivo*. The trend of cytokine responses to the synthetic CII peptide were similar in spleen cells and lymph node cells (Fig 4H). Production of IL-1β, IL-6 and TNFα in response to synthetic CII peptide was significantly inhibited in cells isolated from the CII-immunized HLA-DR1 mice treated with Gö6976, but not in cells isolated from the mice treated with Gö6983, compared to those in cells isolated from mice treated with vehicle. IFNγ and IL-17A production in response to synthetic CII peptides was significantly suppressed in cells isolated from the CII-immunized HLA-DR1 mice treated with Gö6976 or Gö6983.

Levels of serum anti-CII total IgG and anti-CII IgG2a at day 55 post CII-immunization were significantly reduced in CII-immunized HLA-DR1 mice treated with Gö6976 compare to the vehicle-treated control CIA mice (Fig 4I and 4K). In contrast, there was no significant reduction in serum levels of anti-CII total IgG and anti-CII IgG2a antibodies in CII-immunized HLA-DR1 mice treated with Gö6983 compare to the vehicle-treated control CIA mice (Fig 4I and 4K). Although there was a tendency toward reduction in anti-CII IgG2b antibody levels in the Gö6976-treated group compared to levels of anti-CII IgG2b antibodies in the vehicle-treated group or Gö6983-treated group, the difference was not statistically significant (Fig 4L). The levels of serum anti-CII IgG1 were comparable in all three groups (Fig 4J).

Analysis of histopathological features of arthritis in joints in these mice also showed that CII-immunized HLA-DR1 mice treated with Gö6976 were significantly protected from joint damage (Fig 4M and 4N). In contrast, CII-immunized HLA-DR1 mice treated with Gö6983 showed substantial leukocyte infiltration in joint spaces and severe pannus formation, cartilage damage, and bone erosion. The degree of joint damage in Gö6983-treated mice was comparable to that in vehicle-treated control mice. Taken together, our results showed that Gö6976 treatments significantly suppress joint inflammation and development and progress of CIA. Our results also indicate that PKDs that can be inhibited by Gö6976 (presumably PKD1 activated through the TLR/IL-1R signaling pathway) play a pivotal role in the development and progression of CIA and that successful inhibition of arthritis development with a PKD inhibitor at the early stage might be beneficial to delay (or halt) the progression of arthritis.

### Ameliorating effects of Gö6976 on CIA may be due to its inhibitory effects on TLR-mediated activation of immune cells

**Fig 5:**
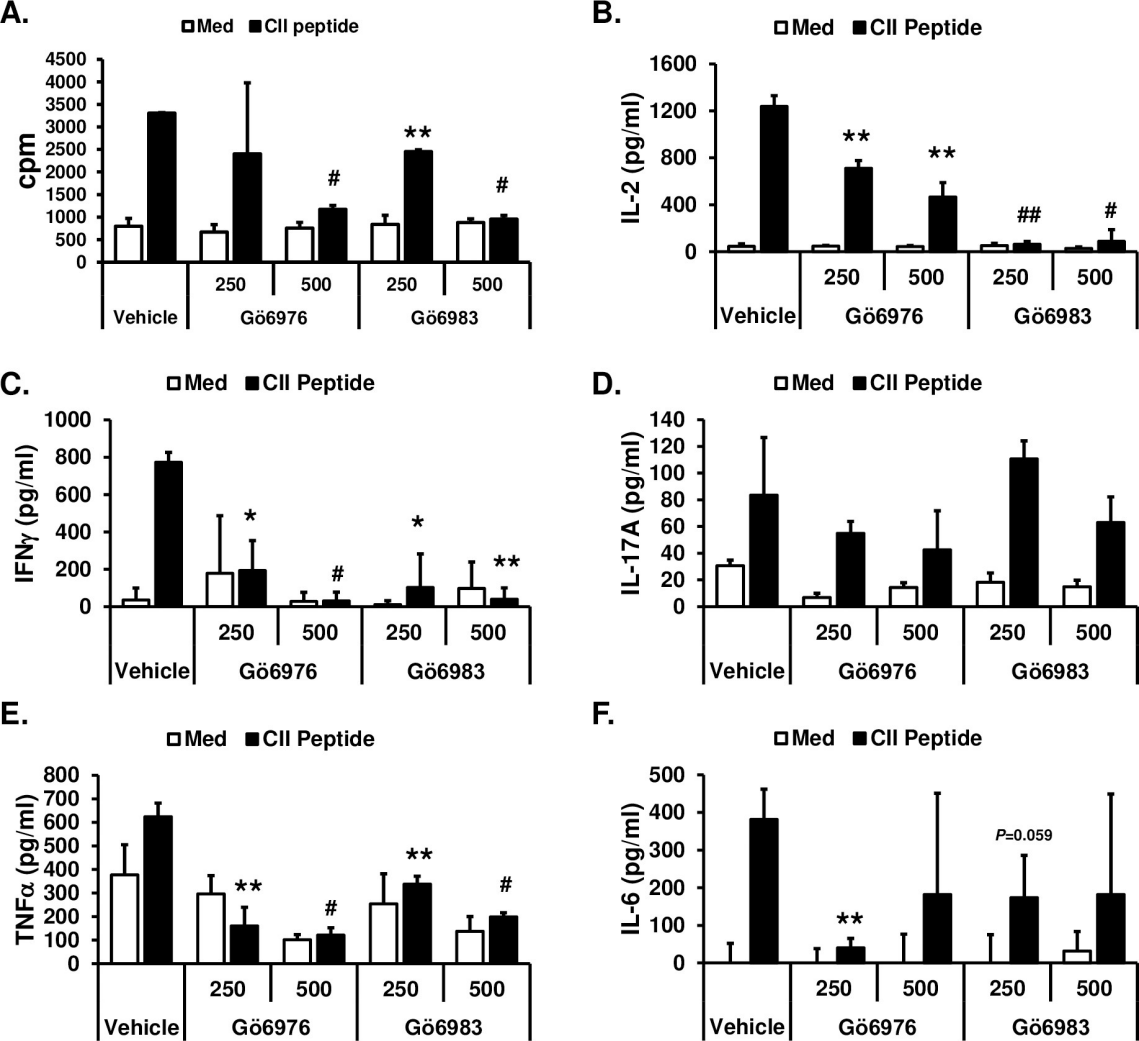
Inhibitory effects of Gö6976 and Gö6983 on TCR-mediated T cell activation. Spleen cells were isolated from humanized CII-specific TCR/HLA-DR1 double transgenic mice. **(A)** Cells (1x10^5^ cells/300 μl/well in 96-well plate) were pretreated with vehicle (1% v/v DMSO), Gö6976 (250 ng/ml or 500 ng/ml), or Gö6983 (250 ng/ml or 500 ng/ml) for 30 min and then stimulated with media or synthetic CII peptide (10 μg/ml) for 48 hrs. One μCi of [^3^H]thymidine was added to each well, and the cells were harvested after an additional 16 hr of culture. Data present the mean (cpm) ± SD of triplicates. **(B-F)** Cells (4x10^5^ cells/200 μl/well in 96-well plate) were pretreated with vehicle (1% v/v DMSO), Gö6976 (250 ng/ml or 500 ng/ml), or Gö6983 (250 ng/ml or 500 ng/ml) for 30 min and then stimulated with media or synthetic CII peptide (50 μg/ml) for 48 hrs. The levels of the indicated cytokine in the culture supernatants were measured by ELISA. Data represent the mean concentration (pg/ml) ± SD of triplicates. The differences between the control (vehicle pretreated group) and experimental group (Gö6976 or Gö6983 pretreated group) were evaluated using two-tailed Student’s *t*-test. *, *p* < 0.05; **, *p* < 0.005; #, *p* < 0.0005; ##, *p* < 0.00005. Experiments were repeated three to five times with similar results.

We further investigated whether the ameliorating effects of Gö6976 on CIA are due to its effects on T cells by interfering with TCR-mediated T cell activation and/or its effects on immune cells by suppressing the TLR-mediated activation of cells. To investigate whether Gö6976 and Gö6983 differentially affect TCR-mediated T cell activation, spleen cells isolated from humanized CII-specific TCR/HLA-DR1 double transgenic mice were stimulated with synthetic CII peptide (specific antigen for TCR in TCR/HLA-DR1 dtg) in the presence or absence of Gö6976 or Gö6983. As shown in Fig 5, synthetic CII peptide induced T cell proliferation and production of IL-2, IFNγ, TNFα, and IL-6. IL-17A production by T cells after synthetic CII peptide stimulation was insignificant (Fig 5D). Although both Gö6976 and Gö6983 significantly inhibited the synthetic CII peptide-induced IL-2 production, Gö6983 inhibited the synthetic CII peptide-induced IL-2 production more effectively than Gö6976 (Fig 5B). Both Gö6976 and Gö6983 inhibited the synthetic CII peptide-induced cell proliferation and production of IFNγ, TNFα, and IL-6 to a similar degree (Fig 5C, 5E and 5F). These results indicate that both Gö6976 and Gö6983 similarly inhibit TCR-mediated activation of T cells.

**Fig 6:**
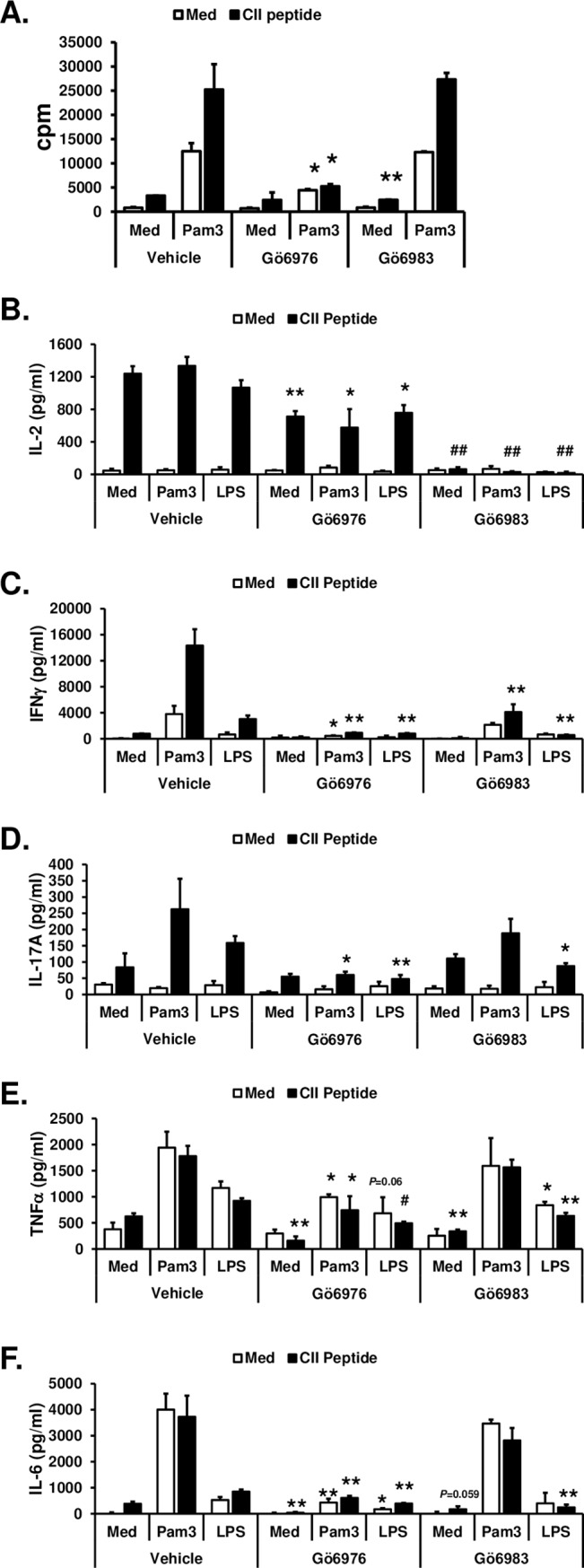
Differential effects of Gö6976 and Gö6983 on TLR-mediated spleen cell proliferation and cytokine production. Spleen cells were isolated from humanized CII-specific TCR/HLA-DR1 double transgenic mice. **(A)** Cells (1x10^5^ cells/300 μl/well in 96-well plate) were pretreated with vehicle (1% v/v DMSO), Gö6976 (250 ng/ml), or Gö6983 (250 ng/ml) for 30 min and then stimulated with media or Pam_3_CSK_4_ (Pam3; 500 ng/ml) in the presence or absence of synthetic CII peptide (10 μg/ml) for 48 hrs. One μCi of [^3^H]thymidine was added to each well, and the cells were harvested after an additional 16 hr of culture. Data present the mean (cpm) ± SD of triplicates. **(B-F)** Cells (4x10^5^ cells/200 μl/well in 96-well plate) were pretreated with vehicle (1% v/v DMSO), Gö6976 (250 ng/ml), or Gö6983 (250 ng/ml) for 30 min and then stimulated with media, Pam_3_CSK_4_ (Pam3; 500 ng/ml), or LPS (25 ng/ml) in the presence or absence of synthetic CII peptide (50 μg/ml) for 48 hrs. The levels of the indicated cytokine in the culture supernatants were measured by ELISA. Data represent the mean concentration (pg/ml) ± SD of triplicates. The differences between the control (vehicle pretreated group) and experimental group (Gö6976 or Gö6983 pretreated group) were evaluated using two-tailed Student’s *t*-test. *, *p* < 0.05; **, *p* < 0.005; #, *p* < 0.0005; ##, *p* < 0.00005. Experiments were repeated three to five times with similar results.

Next, to investigate whether Gö6976 and Gö6983 differentially affect the TLR-mediated activation of immune cells in the presence or absence of TCR signaling, spleen cells isolated from CII-specific TCR/HLA-DR1 dtg were stimulated with a TLR ligand [the TLR2 ligand Pam_3_CSK_4_ (Pam3) or the TLR4 ligand LPS] or TLR ligand plus synthetic CII peptide in the presence or absence of Gö6976 or Gö6983. Fig 6A showed that Pam3 induced splenic cell proliferation and that Pam3 and synthetic CII peptide synergistically induce splenic cell proliferation. Both Pam3-induced cell proliferation and synergistic induction of splenic cell proliferation by Pam3 and synthetic CII peptide were significantly inhibited by Gö6976, while Gö6983 showed no effect on the splenic cell proliferation induced by either Pam3 or Pam3 plus synthetic CII peptide. As shown in Fig 6B, Pam3 and LPS did not induce IL-2 production. Pam3 and LPS also did not have any effect on synthetic CII-induced IL-2 production. Gö6976 significantly, but partially, inhibited synthetic CII peptide-, Pam3 plus synthetic CII peptide-, and LPS plus synthetic CII peptide-mediated IL-2 production. Gö6983 ablated synthetic CII peptide-, Pam3 plus synthetic CII peptide-, and LPS plus synthetic CII peptide-mediated IL-2 production. Fig 6C showed that Pam3 (*p* = 0.0065) and LPS (*p* = 0.027) significantly induced IFNγ production in spleen cells. Pam3-mediated IFNγ production was significantly inhibited by Gö6976. LPS-mediated IFNγ production was substantially, but not significantly, inhibited by Gö6976. In contrast, Gö6983 did not inhibited IFNγ production induced by Pam3 or LPS. Levels of IFNγ were significantly higher in Pam3 plus synthetic CII peptide- and LPS plus synthetic CII peptide-stimulated spleen cells compared to those in each stimulus alone-treated spleen cells. Both Gö6976 and Gö6983 significantly inhibited Pam3 plus synthetic CII peptide- and LPS plus synthetic CII peptide-mediated IFNγ production in spleen cells. Interestingly, levels of IFNγ production after TLR ligand plus synthetic CII peptide stimulation in the presence of Gö6983 were comparable to the levels of IFNγ production after TLR ligand alone stimulation. There was no significant increases in IL-17A production after synthetic CII peptide, Pam3, or LPS stimulation (Fig 6D). However, levels of IL-17A production were significantly higher in Pam3 plus synthetic CII peptide-stimulated cells (*p* = 0.039) and in LPS plus synthetic CII peptide-stimulated cells (*p* = 0.053) compared to those in synthetic CII peptide-stimulated cells. These enhancing effects on IL-17A production by Pam3 and LPS were abolished in the presence of Gö6976 and partially reduced in the presence of Gö6983. Both Pam3 and LPS induced production of TNFα and IL-6 in spleen cells (Fig 6E and 6F). Addition of synthetic CII peptide did not enhance TLR ligand-induced production of TNFα and IL-6 in spleen cells. Pam3- and Pam3 plus synthetic CII peptide-mediated productions of TNFα and IL-6 were significantly inhibited by Gö6976, but not by Gö6983. LPS-mediated production of IL-6 was also significantly inhibited by Gö6976, but not by Gö6983 (Fig 6F). LPS- and LPS plus synthetic CII peptide-mediated productions of TNFα and LPS-plus synthetic CII peptide-mediated IL-6 production were significantly, but partially, inhibited by either Gö6976 or Gö6983 (Fig 6E and 6F). Taken together, our results suggest that Gö6976, but not Gö6983, inhibits TLR-mediated activation of immune cells, while both Gö6976 and Gö6983 similarly inhibit TCR-mediated activation of T cells. Our results imply that ameliorating effects of Gö6976 on the development and progression of CIA are, in part, due to its ability to block the TLR/IL-1R-mediated production of proinflammatory mediators in immune cells.

## Discussion

In the present study, we investigated whether PKD1 can be activated by TLR/IL-1R ligands and contribute to the TLR/IL-1R-mediated proinflammatory responses in HFLS and whether development and progress of CIA can be ameliorated by prophylactic treatment with Gö6976, a pharmacological agent that inhibits PKD.

In addition to immune cells, fibroblast-like synoviocytes (FLS) play a key role in the pathogenesis of RA [35–37]. FLS can be activated by microbial or endogenous TLR ligands and proinflammatory cytokines, and produce various proinflammatory mediators that are involved in cell-cell communication among the FLS and immune cells and contribute to the positive feedback and bone destruction in the affected joints [35, 38, 39]. Previous studies demonstrated that TLR/IL-1R signaling adaptor MyD88 is essential for spontaneous production of proinflammatory cytokines and MMPs by RA synovial cells [13]. PKD1 is activated via all TLR/IL-1Rs that utilize MyD88 as a signaling adaptor, and is essential for the MyD88-dependent TLR/IL-1R-induced expression of proinflammatory mediators in immune cells in human and mouse [17]. Therefore, it is possible that activity of PKD1 is altered in RA synoviocytes and related to the joint inflammation status. Similar to what we have seen in immune cells [17–21], our results show that PKD1 can be activated in human primary FLS (HFLS-N and HFLS-RA) in response to TLR/IL-1R ligands, and that PKD1 plays an essential role in the TLR/IL-1R-mediated expression of proinflammatory mediators in both HFLS-N and HFLS-RA. Our results also show that a pharmacological agent that inhibits PKD can suppress TLR/IL-1R-mediated expression of cytokines and chemokines in HFLS-RA. Considering a role for both immune cells and FLS in joint inflammation and RA, our results argue in favor for our hypothesis that PKD1 is one of the critical factors for development of proinflammatory reactions in the joints in susceptible individuals, and that PKD1 might play a critical role in exaggerated proinflammatory responses in synoviocytes in RA patients.

Freshly isolated HFLS-RA retain an activated phenotype in *ex vivo* for several weeks and can be reactivated by inflammatory cytokines, such as IL-1 [40]. Although HFLS-RA used in this study showed the higher levels of PKD1 phosphorylation in unstimulated, as well as TLR/IL-1R ligand-stimulated, condition compared to those in HFLS-N used in this study, this does not necessarily indicate that PKD1 is hyper-activated in FLS in RA because a single patient sample from each group were utilized in this study. The relation between PKD1 and human RA is currently unknown. It is of great interest whether levels of PKD1 activation and/or expression correlate with the severity and progression of human RA, as well as the magnitude of inflammatory responses in the effected joints of RA patients, and whether there is any single-nucleotide polymorphism in PKD1 related to arthritis susceptibility or severity. Further investigations regarding these aspects are warranted.

Considering the role of PKD1 in TLR/IL-1R signal transduction and proinflammatory responses, and contributions of TLR/IL-1R on experimental inflammatory arthritis and human RA, a pharmacological agent that can inhibit function of PKD1 might be a reasonable new candidate as a disease-modifying drug for RA. Previously, we and others have found that Gö6976 effectively blocks proinflammatory responses induced by several TLR ligands [17, 18, 41, 42]. We have also found that in contrast to Gö6976 (inhibiting PKCα, PKCβ, and PKD), Gö6983 (inhibiting conventional PKCs, PKCδ and PKCζ, but not PKD) has little or no effect on TLR-mediated proinflammatory responses [18, 43]. Subsequently, we found that the inhibitory effect of Gö6976 on TLR/IL-1R-mediated proinflammatory responses is due to its ability to inhibit activity of PKD1 [17, 18, 21]. In addition to inhibiting PKD1 activation and subsequent production of proinflammatory cytokines in response to synthetic or purified TLR/IL-1R ligands *in vitro*, Gö6976 has also been shown to effectively inhibit whole bacteria-mediated proinflammatory responses *in vivo* by inhibiting PKD1 activation occurring through MyD88-dependent TLRs and bacterial interactions [19, 20]. Currently, efficacy of Gö6976 on chronic inflammatory diseases in which TLRs/IL-1Rs play a pathogenic role, like RA, is unknown. We tested effects of Gö6976 on a humanized mouse model of autoimmune arthritis that is based on mice that are transgenic for the RA susceptibility [HLA alleles DRB1*0101 (DR1)] and their susceptibility to autoimmune arthritis when immunized with CII [23]. Our results showed that daily treatment of Gö6976 significantly delays the onset and ameliorates severity of the CIA. Previous studies have demonstrated that IgG2a isotype anti-CII antibodies play a critical role in the pathogenesis of CIA and that a shifting from a Th1 to Th2 profile leads to a clinical improvement of CIA [44–47]. Gö6976 treatment also significantly reduces levels of circulating IgG2a isotype anti-CII antibody in CII-immunized mice. Although there is no significant changes in IgG1 isotype anti-CII antibody levels in CII–immunized mice by Gö6976 treatment, the ratio of IgG1/IgG2a is slightly increased in the Gö6976-treated group (1.08 to 1.204). Our results indicate that the reduced humoral response to CII by Gö6976 treatment is associated with a suppressed Th1-type inflammatory responses without promoting Th2-type response.

In addition to PKD1, Gö6976 inhibits PKCα, PKCβ, PKD2, PKD3, and check point kinase (CHK) 1 and 2 [43, 48]. It is not possible to assess in the current study how much of Gö6976 effects on CIA are due to inhibiting PKD1 and how much of these effects are due to inhibiting CHKs, PKCs and/or other PKD family members. CHKs have been known to be activated through DNA damage signaling pathway [49]. In addition to DNA damage, BCR/ABL signaling can activate CHK1 through the PI3K/Akt/GSK3 pathway [50] and Imiquimod (ligand for TLR7/8) can activates CHK1/2 through a TLR7/8-independent ROS generation [51]. In DNA-damaged hematopoietic cells, hematopoietic cytokines, including IL-3 and Erythropoietin, can enhance phosphorylation of CHK1 through the PI3K/Atk/GSK signaling pathway [52]. Activated CHKs can mediates NF-κB and subsequent proinflammatory cytokine production in p53-deficient cells [53]. On the other hand, expression of CHK1 can be modulated by proinflammatory cytokines. IL-6 signaling can lead to upregulation of CHK1 expression [54], while CHK1 expression can be down-regulated by IFNγ [55]. It is currently not known whether CHKs are involved in TLR/IL-1R signaling directly or indirectly and whether CHKs play a role in pathogenesis of chronic inflammation and arthritis. Considering regulatory capacity of CHKs on NF-κB activation pathway and proinflammatory cytokine production as well as contribution of several proinflammatory cytokines (that play an important role on pathogenesis of inflammatory arthritis) on CHK activation and expression, we cannot completely rule out the possibility that a part of the ameliorating effects of Gö6976 on CIA we observed may be at least partially due to its inhibitory effects on CHKs. This possibility is worthy of investigation in the future.

Based on the different specificity of Gö6976 and Gö6983 [43], our results with Gö6983 treatment *in vivo* and *in vitro* imply that certain PKC family members that can be inhibited by Gö6983 (conventional PKCs, PKCδ and PKCζ) also contribute to the inflammatory process during the course of CIA development by playing a role in the Th1/Th17 development and TCR-mediated cell proliferation and cytokine production. However, compared to the effects of Gö6976, the effects of Gö6983 on CIA are less significant. *Ex vivo* responses of spleen cells and lymph node cells isolated from CIA mice to antigenic synthetic CII peptide show that Gö6976 more effectively inhibits production of cytokines (IL-17A, IL-6, IL-1β, and TNFα) that are known to be involved in Th17 development and pathogenesis of RA. In contrast, Gö6983 does not have inhibitory effects on TLR-mediated expression of proinflammatory mediators tested in this study in both mouse spleen cells and HFLS. Collectively, our results suggest that the ameliorating effects of Gö6976 on CIA are at least partially due to its inhibitory effects on TLR-mediated PKD1 activation at the early stage of CIA development. The pharmacological inhibitor that specifically inhibits only PKD1 has yet to be developed. Although they inhibit all three PKD family members, some new small molecule inhibitors for PKD have recently been developed [56]. The efficacies of these new PKD inhibitors for chronic inflammatory diseases where the TLR/IL-1R pathway contributes to the pathogenesis, including RA, might be interesting enough to evaluate in the near future. In addition, the role of PKD1 on the pathogenesis of CIA and other experimental arthritis are currently under investigation using inducible PKD1-gene deficient mice. The current study has been focused on prophylactic effects of Gö6976 on CIA. Further investigations on whether Gö6976 or other newly-developed PKD inhibitors have therapeutic effects on arthritis, whether PKD inhibitors can enhance therapeutic efficacy of the currently existing arthritis treatment regimens, and whether PKD inhibitors have prophylactic and/or therapeutic effects on other experimental models of arthritis, will all be worthy of pursuing in the future. Although more studies are necessary to sort those issues out, our results support the notion that PKD1 might be a reasonable new target for therapeutic intervention for arthritis, and that pharmacological inhibitors of PKD can be useful therapeutics for inflammatory arthritis, including RA. Our results also suggest the necessity for the development of the more specific PKD1 inhibitors that can control exaggerated TLR/IL-1R signaling sequences in chronic inflammatory diseases.

In summary, we have demonstrated that TLR and IL-1R ligands induce activation of PKD1 in HFLS isolated from donors with or without RA, and that PKD1-knockdown or inhibition of PKD by Gö6976 treatment substantially inhibits TLR/IL-1R-mediated proinflammatory cytokine/chemokine expression in HFLS-RA. Daily treatments with Gö6976 significantly delay onset, incidence, and severity of CIA in arthritis-susceptible humanized DR1 tg mice. Collectively, our findings imply that the ameliorating effects of Gö6976 on CIA may be due to its ability to inhibit TLR/IL-1R-activated PKD1 that might play an important role in proinflammatory responses in arthritis, and that PKD1 could be a therapeutic target for inflammatory arthritis.

## Supporting information

S1 Fig Uncropped gel and blot scans. **(A)** Uncropped blots for Fig 1A and densitometric quantitation of each band in the blot. **(B)** Uncropped blots for Fig 1B. **(C)** Repeat experiments for Fig 1B. HFLS-N and HFLS-RA were stimulated with media (med), TLR2 ligand peptidoglycan (PGN; 10 μg/ml), or human recombinant IL-1β (10 ng/ml) for 45 min. Protein levels of actin and phosphorylation status of PKD were detected by Western blot. The density of phosphor-PKD band in each sample was quantitated by densitometry and normalized to the density of the actin band in the same sample. Data represent the fold induction from the normalized densitometric value of phosphor-PKD band of the media-treated HFLS from a normal donor. **(D)** Repeat experiments for Fig 1B. HFLS-N and HFLS-RA were stimulated with media (med), TLR2 ligand Pam3Csk4 (Pam3; 500 ng/ml), or human recombinant IL-1β (10 ng/ml) for 45 min. Protein levels of actin and phosphorylation status of PKD were detected by Western blot. The density of phosphor-PKD band in each sample was quantitated by densitometry and normalized to the density of the actin band in the same sample. Data represent the fold induction from the normalized densitometric value of phosphor-PKD band of the media-treated HFLS from a normal donor. **(E)** Uncropped gels for Fig 1C. **(F)** Uncropped gels for Fig 1D. **(G)** Uncropped gels and blots for Fig 1E. **(H)** Uncropped gels for Fig 1F.
(PDF)
