# Supplementary figures and images for: Ameliorating effects of Gö6976, a pharmacological agent that inhibits protein kinase D, on collagen-induced arthritis
Source: PLoS One. 2019 Dec 6;14(12):e0226145. doi: 10.1371/journal.pone.0226145 (PMC6897462; doi:10.1371/journal.pone.0226145)

A.

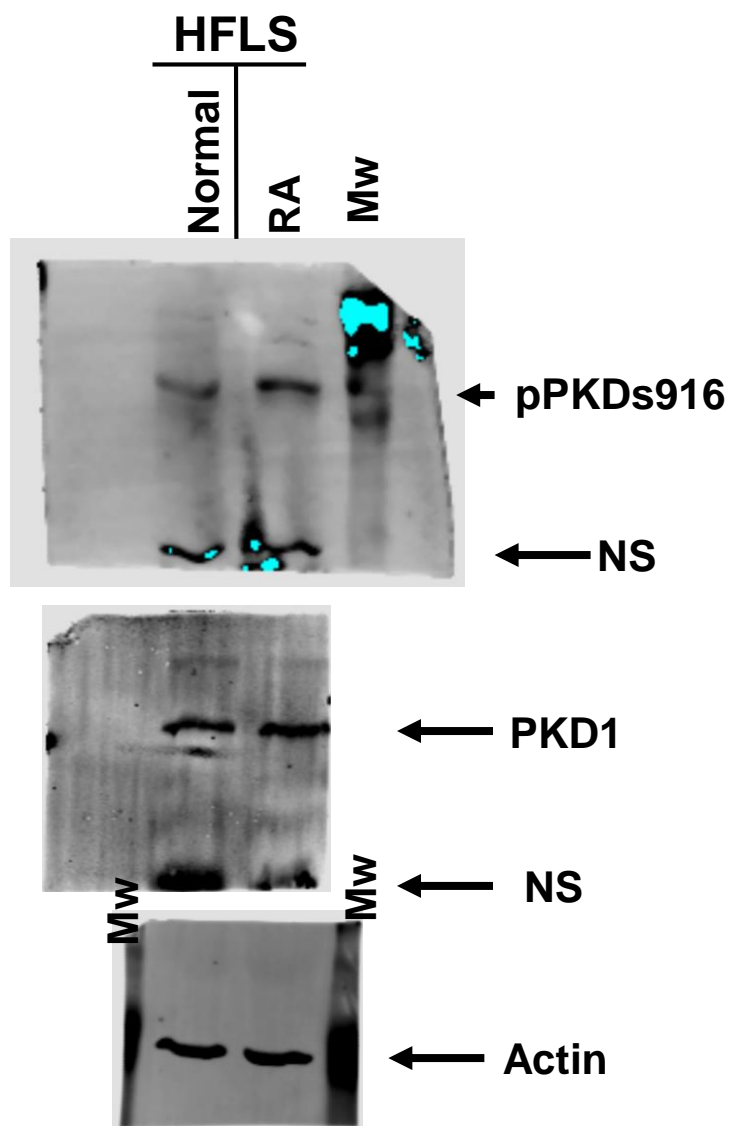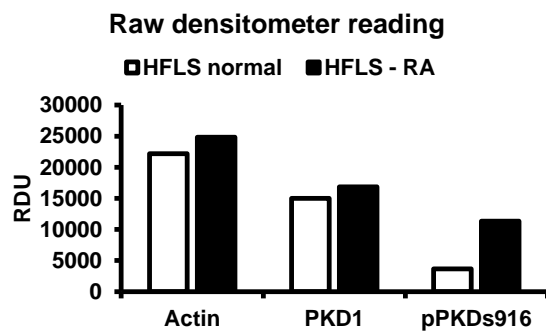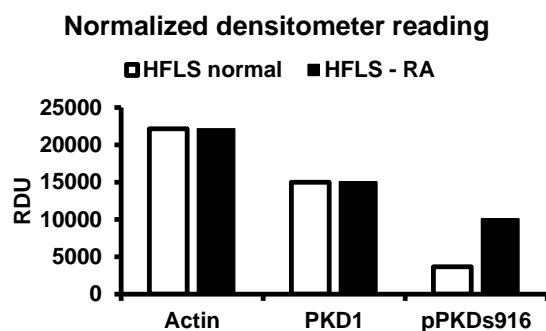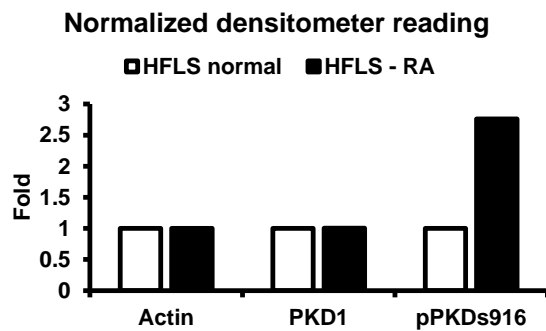

**B.**

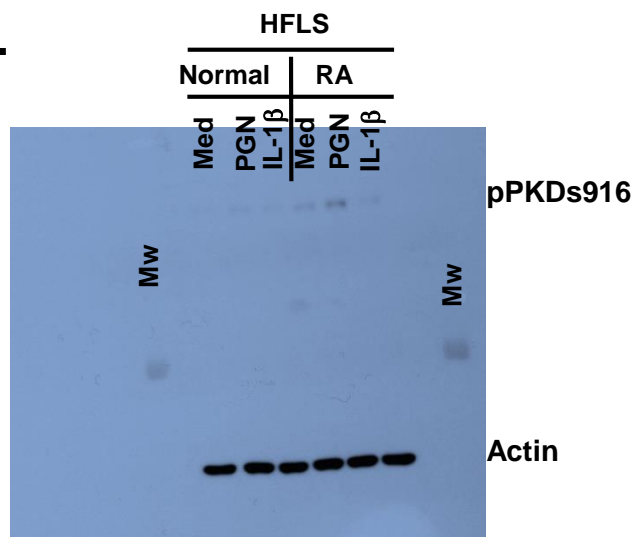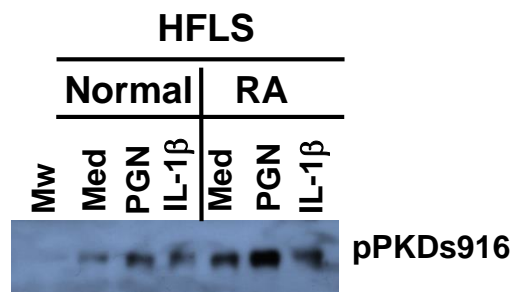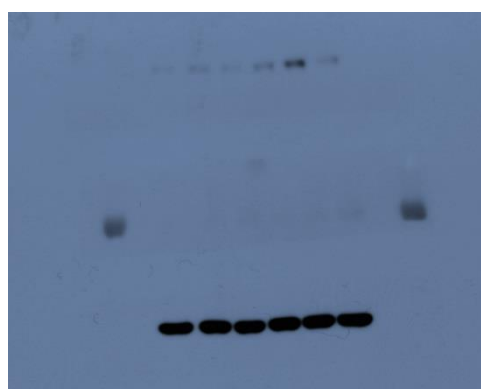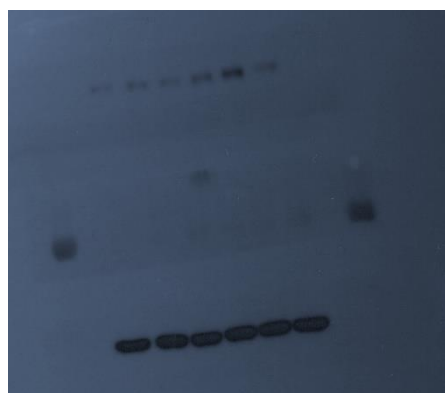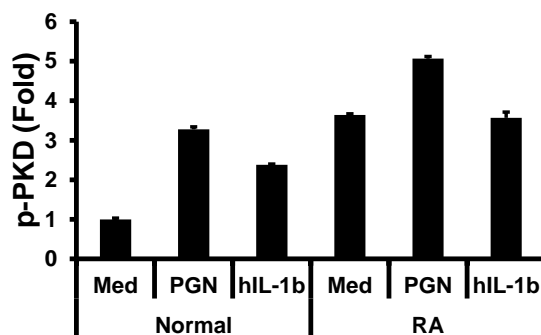

C.

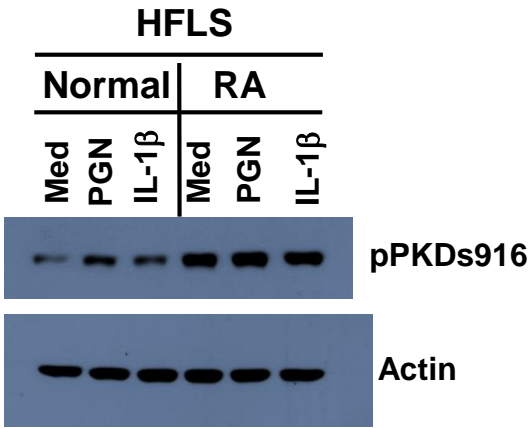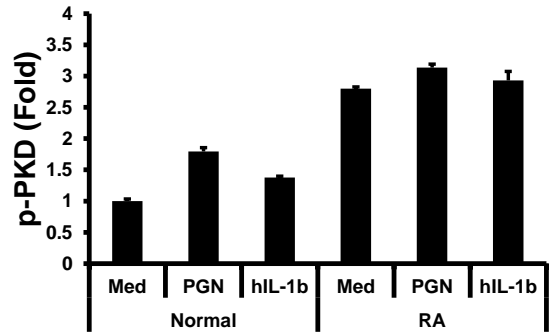

D.

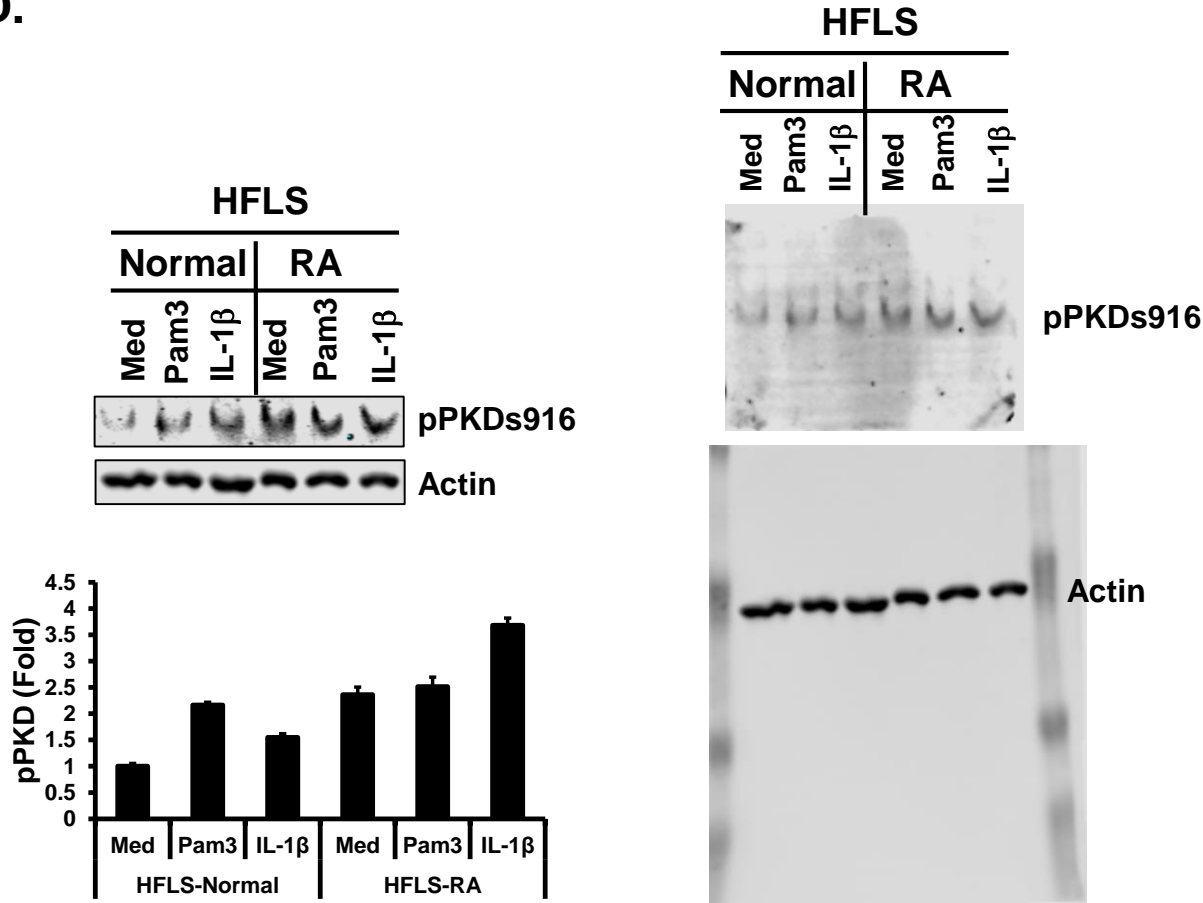

E.

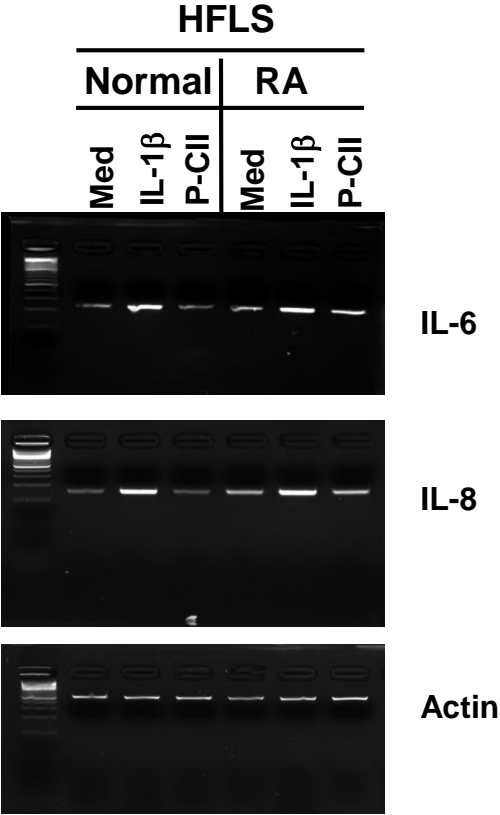

**F.**

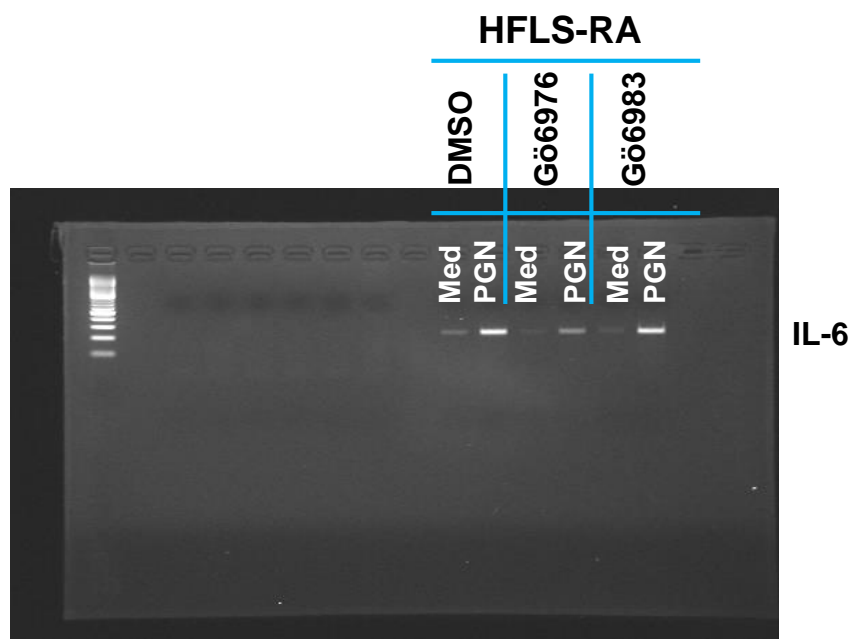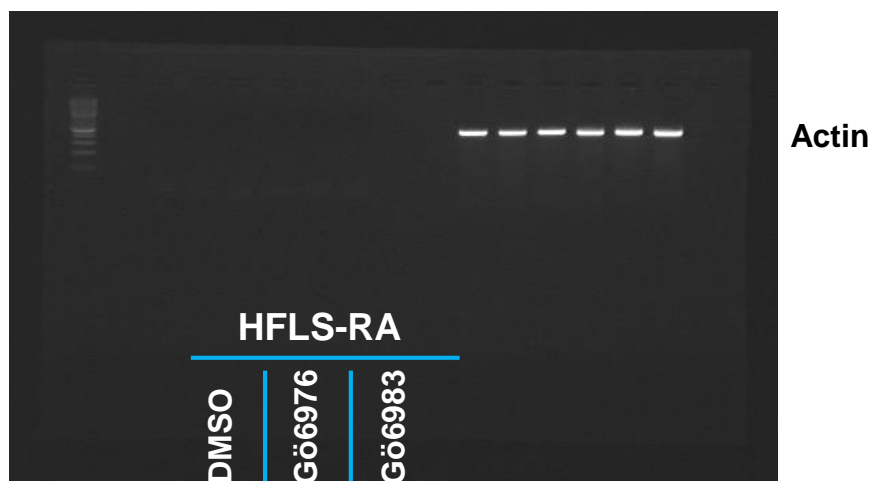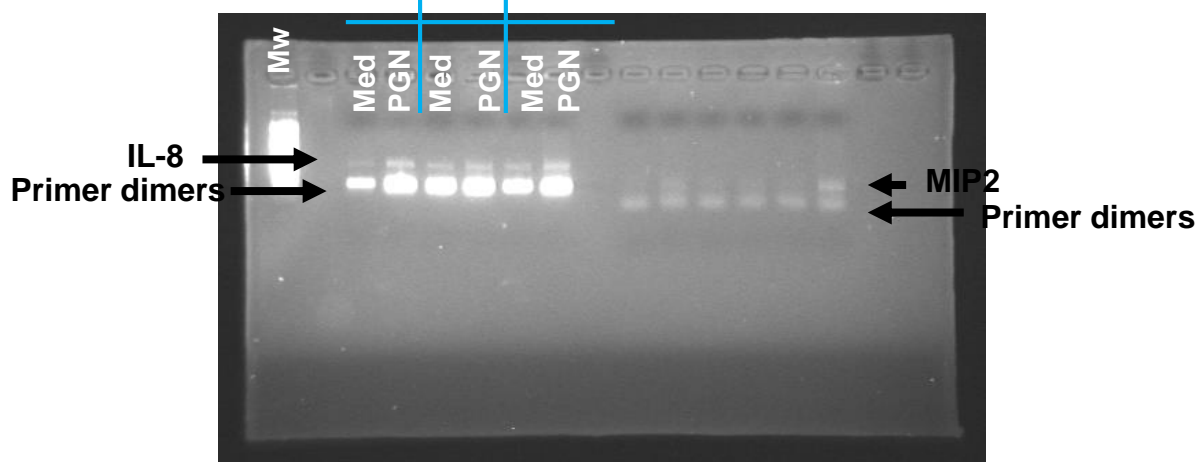

G.

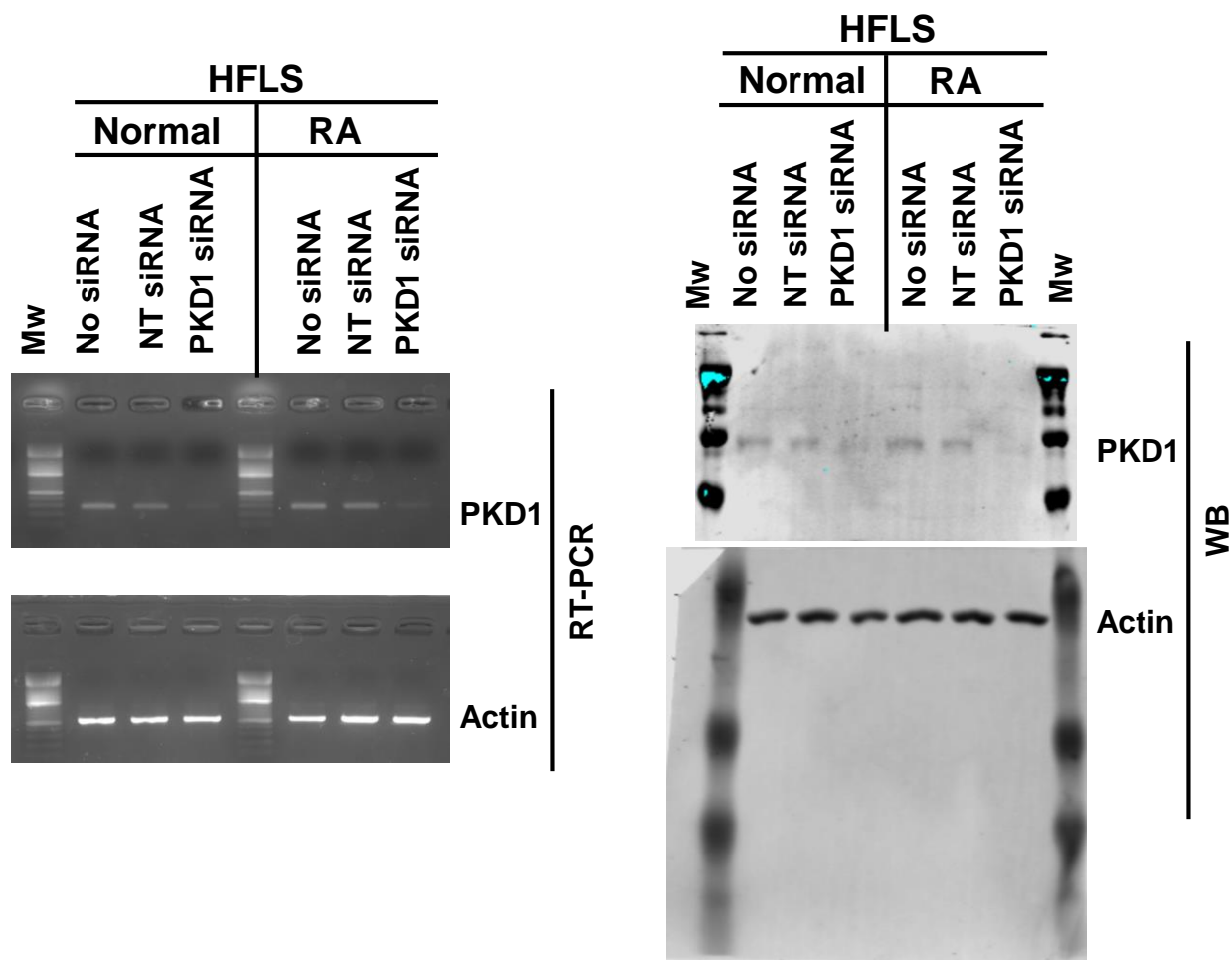

H.

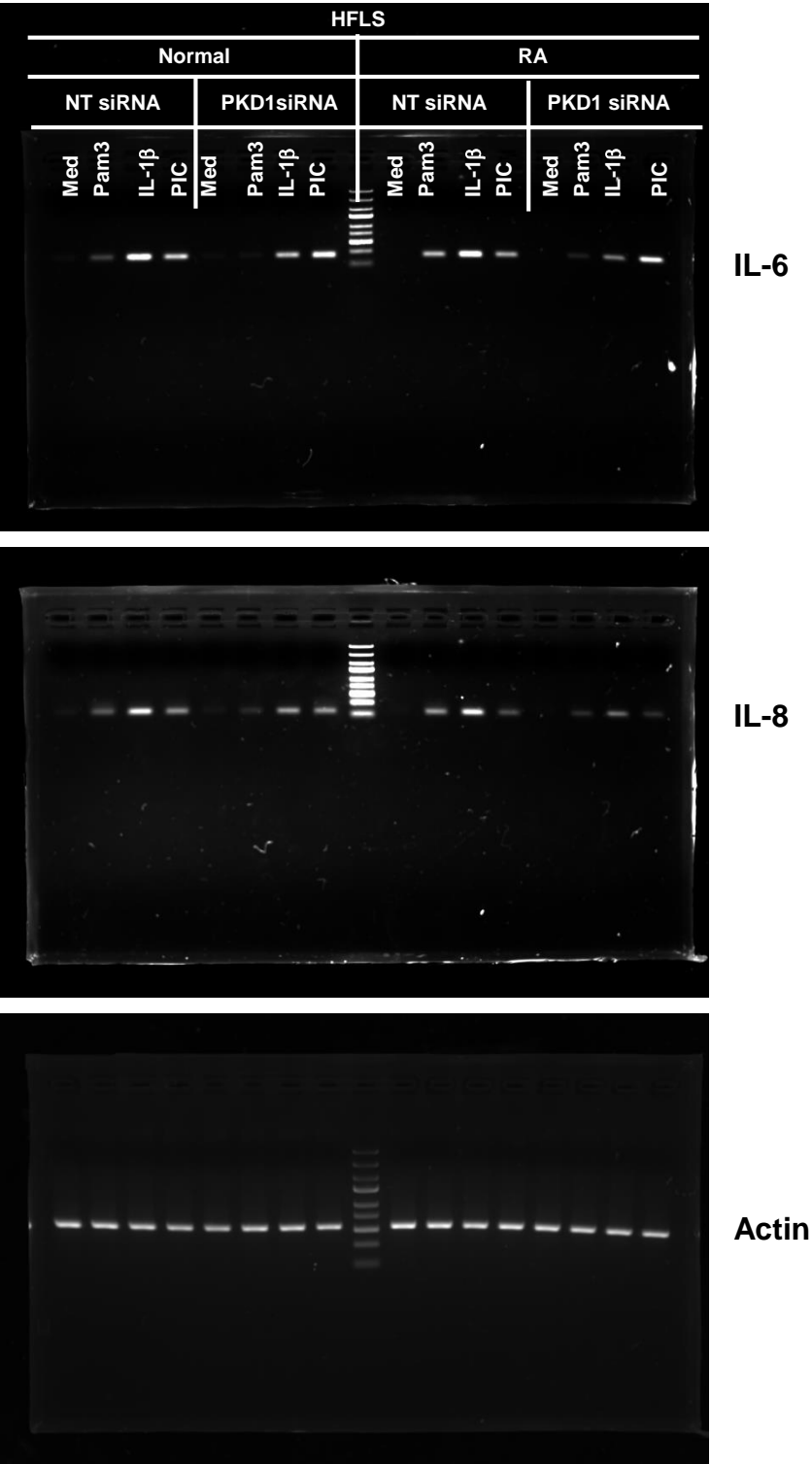

Supplement: S1 Fig — (A) Uncropped blots for Fig 1A and densitometric quantitation of each band in the blot. (B) Uncropped blots for Fig 1B. (C) Repeat experiments for Fig 1B. HFLS-N and HFLS-RA were stimulated with media (med), TLR2 ligand peptidoglycan (PGN; 10 μg/ml), or human recombinant IL-1β (10 ng/ml) for 45 min. Protein levels of actin and phosphorylation status of PKD were detected by Western blot. The density of phosphor-PKD band in each sample was quantitated by densitometry and normalized to the density of the actin band in the same sample. Data represent the fold induction from the normalized densitometric value of phosphor-PKD band of the media-treated HFLS from a normal donor. (D) Repeat experiments for Fig 1B. HFLS-N and HFLS-RA were stimulated with media (med), TLR2 ligand Pam3Csk4 (Pam3; 500 ng/ml), or human recombinant IL-1β (10 ng/ml) for 45 min. Protein levels of actin and phosphorylation status of PKD were detected by Western blot. The density of phosphor-PKD band in each sample was quantitated by densitometry and normalized to the density of the actin band in the same sample. Data represent the fold induction from the normalized densitometric value of phosphor-PKD band of the media-treated HFLS from a normal donor. (E) Uncropped gels for Fig 1C. (F) Uncropped gels for Fig 1D. (G) Uncropped gels and blots for Fig 1E. (H) Uncropped gels for Fig 1F. (PDF) [file pone.0226145.s001.pdf]
